# Midterm follow up in patients with reduction ascending aortoplasty

**DOI:** 10.1186/1749-8090-9-120

**Published:** 2014-07-05

**Authors:** Arndt H Kiessling, Eva Odwody, Alexandra Miskovic, Ulrich A Stock, Andreas Zierer, Anton Moritz

**Affiliations:** 1 Department of Thoracic and Cardiovascular Surgery, Johann Wolfgang Goethe University, Theodor Stern Kai 7, Frankfurt am Main 60590, Germany

**Keywords:** Reduction ascending aortoplasty, Aneurysm, Aorta, Follow-up study, Predictor

## Abstract

**Background:**

The reduction ascending aortoplasty in patients with an aortic ectasia/dilatation is a common procedure during concomitant cardiac operations. Aim of the follow up study was the evaluation of possible re-dilatation and complications.

**Methods:**

From 1998 to 2010 124 patients (69% male; mean age 66.6 ± 12 ys) with ectasia of the ascending aortic who had no further indication for an aortic replacement, were included. The mean preoperative diameter of the ascending aorta was 4.2 ± 0.6 cm. The patients risk profile was moderate (mean EF 51% ± 11%, Euroscore 4.2 ± 2.1). To treat the dilatation of the ascending aorta, a longitudinal incision was performed and a strip of the aortic wall was resected. A reduction aortoplasty was carried out with a double-layered suture line using a 4/0 Prolene mattress suture with an additional 4/0 Prolene running suture. A follow up (rate 95%) was performed by echocardiography- and clinical examination.

**Results:**

All patients underwent reduction aortoplasty associated with a primary cardiac surgical procedure (AVR 63%, CABG 13%, other or combination 24%). The intrahospital mortality rate was 4%. Four aortic bleeding complications occurred. After a mean postoperative period of 57 ± 39 months, the ascending aortic diameter (3.6 ± 0.6 cm) was still significantly (P < 0.01) reduced. No postoperative aortic-related complications including aortic rupture, dissection and reoperation were observed. In 4 patients, the ascending aorta had re-dilated to the preoperative diameter.

**Conclusion:**

Reduction ascending aortoplasty without external wrapping is a safe procedure with acceptable midterm results in patients with asymptomatic dilatations and concomitant cardiac surgical procedures.

## Background

A number of surgical techniques and materials are currently available for the treatment of aneurysms of the ascending aorta. Choosing the right technique requires careful review of the various factors, such as morphology of the aneurysm, dilatation of the aortic root, an additional aortic defect and the risks associated with surgery [1,2].

If the dilatation affects only the ascending aorta, aortic replacement is the most common procedure and may potentially be combined with aortic valve replacement. This procedure provides good results [2] but is still associated with significant risks. Perioperative mortality and morbidities can vary up to 10% [1-4].

Reduction ascending aortoplasty (RAA) constitutes an alternative to replacing the ascending aorta in patients with ascending aneurysm without involvement of the aortic root [5]. This is a viable procedure which has demonstrated a number of advantages (less invasive as compared to replacement with a Dacron graft, shorter aortic clamping time, lower risk of bleeding) [6-8]. In addition, lower mortalities and morbidities were reported in patients with RAA [9]. The RAA remains, however, a controversial surgical option due to the potential risk of re-dilatation. This is why the procedure is generally limited to patients with a high perioperative risk. [2]. Reduction ascending aortoplasty is often used in older patients with non-dissecting aortic aneurysm, in particular as an added procedure with concomitant cardiosurgical intervention [8,9].

Various techniques of aortoplasty are described in literature. Oftentimes synthetic wrapping is used in addition for external support. Our follow-up study, however, included only patients who underwent surgery with the Robicsek et al technique [7,10] (without external support). This follow-up study was designed to assess medium long-term results of non-Dacron supported RAA procedures and determine the potential predictors for re-dilatation.

## Methods

From January 1998 until 2010, 124 patients underwent reduction ascending aortoplasty associated with dilatation and ectasia of the ascending aorta. From 2011 to 2012 the patients were asked to return for a follow-up echocardiography at the Frankfurt am Main University Hospital. The study had been approved by our institutional ethics committee and the investigator obtained written consent from each patient (decree 184/12 Ethic committee Goethe University, Frankfurt, Germany). The aortoplasty was performed when replacement of the ascending aorta was not indicated for patients (>5 cm) or existing pre-operative concomitant diseases would have considerably increased the risk associated with surgery.

The clinical variables are listed in Table 1. The mean age was 66.6 ± 12 years (min. 22 years/max. 89 years). N = 85 patients were male (69%) and 92.7% in the New York Heart Association functional class > II. In order to determine the pre-operative aortic diameter, we performed transthoracic echocardiography or radiological diagnostics (computer tomography). The mean pre-operative aortic diameter measured 4.2 ± 0.6 cm (range 2.8-5.7 cm). None of the patients presented with Marfan syndrome or other genetic disorders for which aortoplasty would be contraindicated. In all patients, the aortic dilatation was limited to the ascending aorta without involvement of the aortic root or aortic arch. The primary indication for surgery was aortic valve disease in n = 73 (63%) of patients. In n = 16 (13%) of patients, an aortocoronary bypass presented an indication for sternotomy. In addition to the dilatation of the ascending aorta, primary cardiac surgery was indicated in all patients. Reduction ascending aortoplasty was performed as concomitant secondary surgery (Table 2).

**Table 1:** Preoperative parameters

**Items**	**Number or mean ± SD**	**Percent or range**
Age (years)	66 ± 12	22-89
Male	N = 85	69%
Body weight (kg)	76 ± 15	45 – 114
Body height (cm)	171 ± 8.5	152 – 189
Aortic valve disease	N = 78	63%
Bicuspid	N = 39	44%
Coronary heart disease	N = 16	13%
Re-Operation	N = 4	3%
Elective	N = 99	80%
Euroscore I	4.2 ± 2.1	1-15
Aortic ascendens diameter (cm)	4.2 ± 0.6	2.8-5.7

**Table 2:** Perioperative procedures

**Procedure**	**Number or mean ± SD**	**Percent or range**
Concomittent aortic valve replacement	63	63%
Aortic valve reconstruction	15	12%
Partial upper sternotomy	33	27%
Comcomittent mitral valve operation	11	9%
CABG	16	13%
Distal bypass anastomoses	2,1 ± 1,2	1-6
Aortic wall resection (cm)	1.6 ± 0.9	0.6-5

### Surgical technique

After complete longitudinal sternotomy in 63% of all patients (27% underwent partial upper sternotomy), a cardiopulmonary bypass was established under moderate hypothermia (34°C). The reduction ascending aortoplasty consisted of the direct resection of an oval section of the anterior wall of the ascending aorta. The expected reduction was calculated with the Roman formula [11] (circumference = 2πr (r = radius, π = 3.14). The aortotomy was then adjusted in two layers with 4/0 Prolene™ (Ethicon Inc. USA) suture material. The aortoplasty was not additionally supported mechanically with prosthetic material. All associated indications for cardiac surgery were performed before the aortoplasty.

The patient follow-up data was initially collected by means of postal letters, telephone interviews and subsequent transthoracic echocardiography. If a CT of the chest had already been performed after hospitalization, echocardiography was not carried out and the data from the radiology test was used. The cumulative follow-up period consisted of 565 patient years and was concluded at 95% (N = 119/124) for the endpoint “survival”. An echocardiography was performed in 65 patients. The median follow-up period was 55 months and the mean follow-up time was 57 ± 34 months.

### Statistical analysis

Continuous variables were specified as mean value ± standard deviations. Nominal variables were described as numbers and percentages. Life tables were calculated with the Kaplan-Meier method. Significant predictors for a re-dilatation were first examined in a univariate analysis followed by a gradual logistic regression analysis. Only variables which were significant in the univariate analysis were entered into the regression model. A p-value of less than 0.05 was considered to be statistically significant. Statistical analyses were carried out with the SPSS 21.0 software package (SPSS Inc., IBM, NY).

## Results

### Postoperative and perioperative mortality and morbidity

Already during the primary hospitalization, 5 patients (4%) died within the first 30 days after the procedure. The cause of death was low cardiac output syndrome (n = 3) as well as multiple organ failure (n = 2). Acute antegrade dissection was not reported.

### Long-term survival

**Figure 1:**
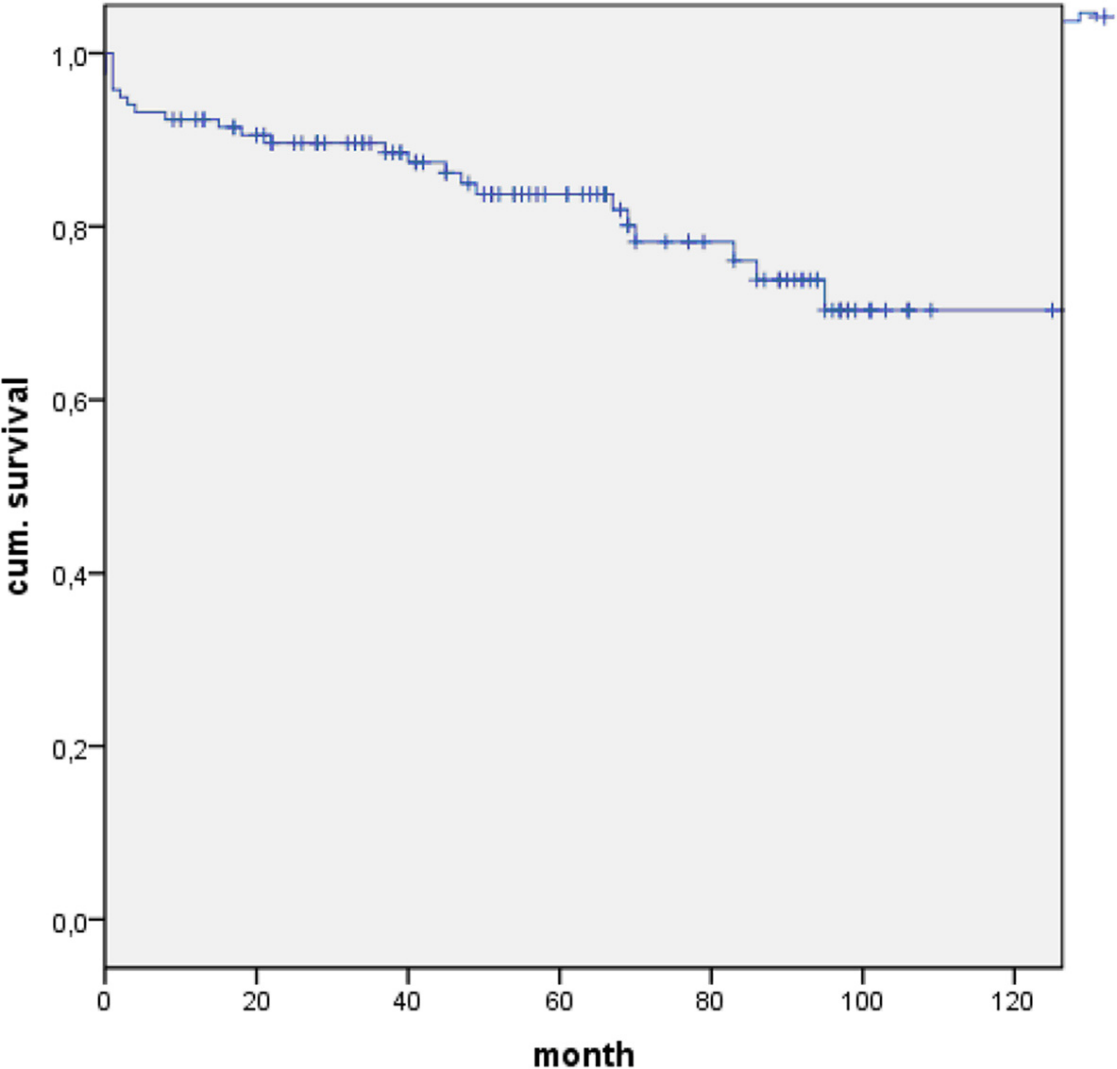
Kaplan Meier Curve for Survival after aortoplasty procedure.

20 cases of late death occurred within the entire follow-up period. The patients’ mean age of death was 72 ± 11 years. The exact causes of death could not be established. No autopsies were performed (Figure 1).

### Complications

4 of 124 patients experienced increased secondary haemorrhaging after surgery which could not be managed despite conservative medicinal therapy. Re-thoracotomy provided evidence of the source of the haemorrhage at the aortoplasty suture line. The bleeding was stopped in all cases with surgical intervention (Table 3).

**Table 3:** Follow up

**Items**	**Number or mean ± SD**	**Percent or range**
Age (years)	72 ± 11	37 – 97
Lost to follow up	5/124	4%
Mortality rate	20	21%
Atrial fibrillation	27	27%
Aortic ascendens diameter (cm)	3.6 ± 0.6	2.3-4.9
Re-operation Aortic site	4	4%
Warfarin therapy	31	31%
NYHA III-IV	8	8%

### Redilatation and repeat surgery

**Figure 2:**
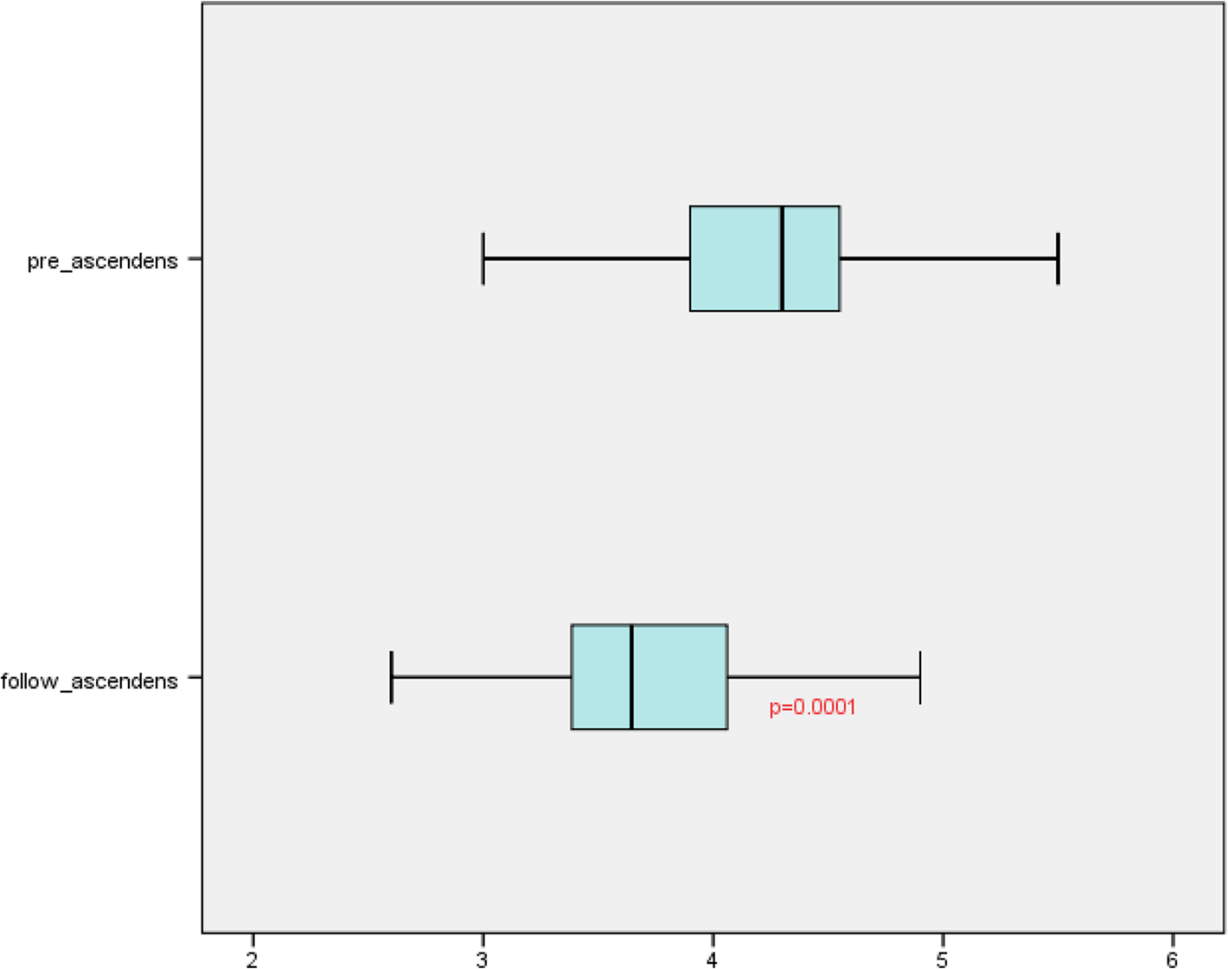
Boxplot of pre- and postoperative mean diameter of aortic roots after aortoplasty.

**Figure 3:**
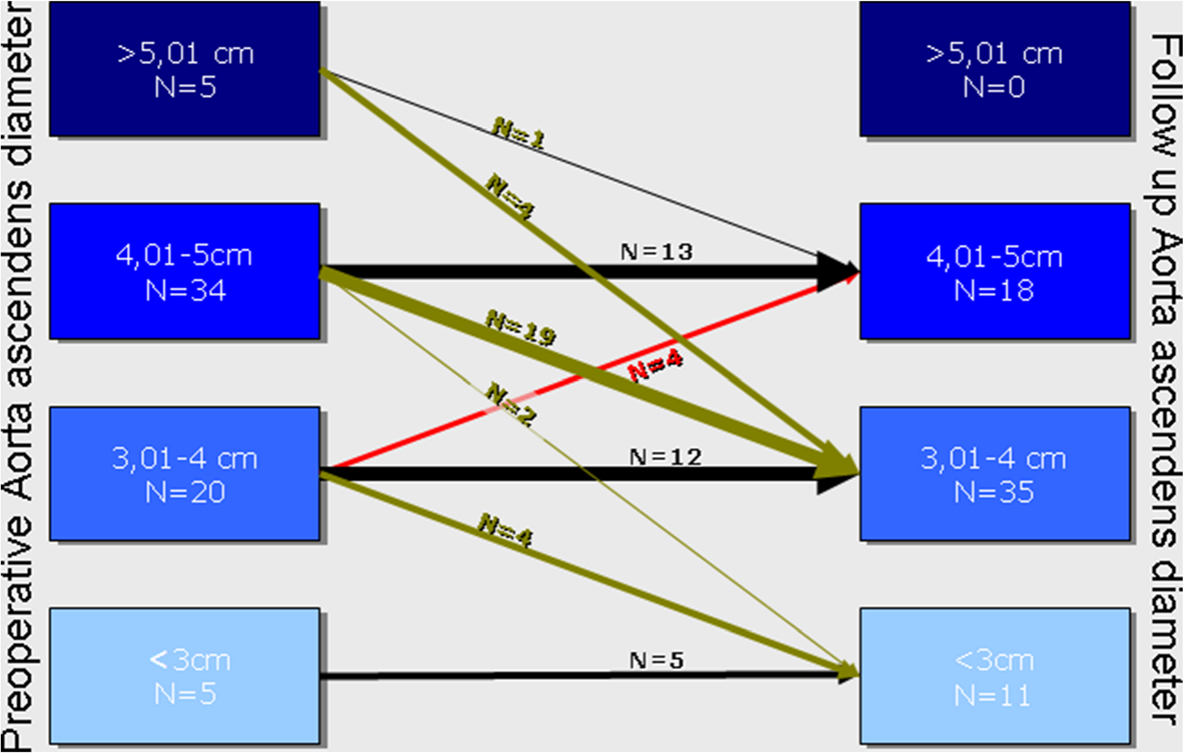
Changes of pre- and postoperative aortic diameters.

With the technique applied for the reduction ascending aortoplasty, the aortic diameter (3.6 ± 0.6 cm range 2.3-4.9 cm) in the area of the ascending aorta was significantly reduced by 0.6 cm (P = 0.001) (Figure 2). Progression of the aortic dilatation occurred in 4 patients (Figure 3). With a baseline diameter of 3.3 ± 0.5 cm, the cross-section increased by an average of 0.9 cm in these patients (4.2 ± 0.7cm). In the remaining patients (94%) the diameter was maintained with the reduction aortoplasty technique (n = 30) or decreased (n = 29). Repeat surgery with the indication of aortic revision were not reported during the follow-up period.

Predictors for redilatation: Only the preoperative diameter was identified as a significant predictor for redilatation. This applied only to patients with a smaller preoperative baseline diameter of <40 mm where redilatation was not observed. Other pre- and intra-operative variables (e.g. surgeon) could not be evaluated as a predisposing factor for redilatation.

## Discussion

The reduction ascending aortoplasty is a minimally invasive therapy option for dilatations of the ascending aorta and a viable alternative to radical aortic replacement. It is generally used for patients with borderline aortic dilatation or increased surgical risks [9]. Reduction aortoplasty can effectively decrease the aortic diameter or prevent further dilatation, shorten the aortic clamping time, and decrease the rate of mortality and morbidity [12,13]. Postoperative complications were mainly caused by the primary indication for surgery (aortic valve replacement or CABG). The good results of the perioperative RAA are in contrast to those of replacement of the ascending aorta. Replacement of the ascending aorta has a higher perioperative mortality and morbidity [4,5,14-16], but is justified by the number of different indications for surgery and patient characteristics [13]. Aortoplasty is contraindicated in patients requiring aortic replacement (type A aortic dissection, Marfan syndrome, cystic medial necrosis). Reduction ascending aortoplasty is generally performed in older patients with a high perioperative risk and in combination with another concomitant heart surgery. The greatest concerns with regard to aortoplasty as surgical treatment pertain to the rate of re-dilatations and mortality. During our follow-up study, 20 patients died. The causes are not known, but in view of concomitant combined cardiac procedures, connections to the underlying cardiac disease can be derived. The results are comparable with those of other studies [2,8,9]. Bauer et al. [13] reports a survival rate of 94% after 5 years and regards the aortoplasty-related mortality as very low. He believes that the reduction ascending aortoplasty has no effect on medium-term or long-term survival.

Literature research on the occurrence of re-dilatation after RAA shows contradictory results with rates of 0% to 25% [12,13,17]. The lack of external support (wrapping) is made responsible for the differences, but a direct comparison between the studies is difficult due to the inhomogeneous groups. Arsan et al. [12] reports 35% of patients with a combined aortic valve replacement as compared to Bauer et al, where 89% of patients received aortic valve replacement with RAA. In our study, the most relevant valvular heart disease was aortic valve replacement associated with relevant stenosis (63%) while Bauer et al. [13] reported a percentage of 47%. Muller et al. [18] also reports that all re-dilatations occurred in patients with Marfan syndrome, while other authors classified Marfan syndrome as an absolute contraindication for RAA [19-21]. We determined an absence of re-dilatation of 94% over a period of 10 years. Four patients had re-dilatation of the ascending aorta which did not require surgery. This result is similar to that of Bauer [13], Kamada [22] and Polvani [17]. There is a general consensus that RAA should not be performed on patients with an aortic aneurysm of more than 60 mm [2]. This is why this patient population is not represented in our population. Univariate and multivariate analyses confirm that the pre-operative diameter is the most important factor for RAA. However, these results mainly applied to a diameter of more than 55 mm [2]. However, our analysis also shows that at the other end of the extreme values, reduction ascending aortoplasty may fail, namely in those patients for whom dilatation of the aorta constitutes a borderline indication for surgery or a normal physiological width.

### Limitations

The validity of this study is very limited. The following aspects should be critically noted: The study was only conducted in a selected subgroup of patients (maximum diameter of 57 mm). There are no control groups where either no surgery was performed on the aorta or aortic replacement was carried out. Histology results on the morphology of the aortic wall were not collected. A 95% follow-up rate with regard to the primary endpoint “death” is acceptable, but only 65% of patients could be motivated to undergo a follow-up echocardiography (re-dilatation as secondary endpoint).

## Conclusion

For our patient population, reduction ascending aortoplasty is a safe and effective treatment option in patients with dilated ascending aorta (<50 mm) or significant contraindications for aortic replacement.

## Competing interests

The authors declare that they have no competing interests.

## Authors’ contributions

AHK carried out the data recruitment, drafted the manuscript and performed the statistical analysis. EO carried out the follow up. AM performed the echo examinations. US, AZ and AM participated in the design of the study and. AM helped to draft the manuscript. All authors read and approved the final manuscript.
